# Host-Pathogen-Treatment Triad: Host Factors Matter Most in Methicillin-Resistant Staphylococcus aureus Bacteremia Outcomes

**DOI:** 10.1128/AAC.01902-17

**Published:** 2018-01-25

**Authors:** M. Cristina Vazquez Guillamet, Rodrigo Vazquez, Benjamin Deaton, Jenny Shroba, Laia Vazquez, Renee-Claude Mercier

**Affiliations:** a Division of Pulmonary, Critical Care, and Sleep Medicine, Department of Internal Medicine, University of New Mexico, Albuquerque, New Mexico, USA; b Division of Internal Medicine, Department of Internal Medicine, University of New Mexico, Albuquerque, New Mexico, USA; c College of Pharmacy, University of New Mexico, Albuquerque, New Mexico, USA; d Department of Internal Medicine, Bridgeport Hospital-Yale University School of Medicine, Bridgeport, Connecticut, USA

**Keywords:** methicillin-resistant Staphylococcus aureus, mortality rates, microbe, host, antibiotic, MRSA, outcomes, treatment

## Abstract

Previous studies have separately emphasized the importance of host, pathogen, and treatment characteristics in determining short-term or in-hospital mortality rates for patients with methicillin-resistant Staphylococcus aureus (MRSA) bloodstream infections. Less is known about the relative importance of these factors and their interactions in determining short-, medium-, and long-term mortality rates. This is an observational cohort study in which data for all patients admitted to the University of New Mexico (UNM) Health Sciences Center (HSC) between July 2002 and August 2013 with MRSA-positive blood cultures were recorded. We collected patients' demographics and treatment data, as well as data on genetic markers of the MRSA isolates. Outcomes of interest were determinants of short-term (within 30 days), medium-term (30 to 90 days), and long-term (>90 days) mortality rates. This study included 273 patients with MRSA bacteremia. Short-, medium-, and long-term mortality rates were 18.7%, 26.4%, and 48%, respectively. Thirty-day mortality rates were influenced by host variables and host-pathogen interaction characteristics. Pitt bacteremia scores, malignancy, and health care exposure contributed to 30- to 90-day mortality rates, while treatment duration of >4 weeks had a protective effect. Age remained a significant risk factor for death at >90 days, while admission leukocytosis was protective. Infection represented the most frequent cause of death for all three time frames; rates varied from 72.6% in the first 30 days and 60% for 30 to 90 days to 35.7% for >90 days (*P* = 0.003). Host characteristics affect short-, medium-, and long-term mortality rates for MRSA bloodstream infections more than do pathogen genetic markers and treatment factors.

## INTRODUCTION

Staphylococcus aureus represents the leading cause of bloodstream infections, with incidence rates of 20 to 50 cases/100,000 population per year and mortality rates as high as 40% (1, 2). Depending on the site of acquisition, methicillin resistance rates have lately decreased, approaching 30%. Infections caused by methicillin-resistant Staphylococcus aureus (MRSA) carry mortality rates twice as high, higher in-hospital complication rates, and greater health care costs, compared with bloodstream infections caused by methicillin-susceptible Staphylococcus aureus (MSSA) (3, 4). Once a hospital-acquired pathogen, MRSA now contributes significantly to community-acquired infections, while also being endemic in many hospitals and nursing homes across the United States.

Although traditionally it has been thought that infectious diseases primarily have short-term effects on patients' health, recent literature shows that long-term, downstream consequences also occur. Other acute infections are joining community-acquired pneumonia in exhibiting long-term effects and, while risk factors for in-hospital death have been studied, less is known about determinants of medium- and long-term mortality rates. Many studies looking at MRSA bacteremia outcomes analyzed the influences of host, pathogen, and treatment factors separately. Age, comorbidities, severity of the acute presentation, and timely treatment, including source control, have consistently been shown to affect outcomes in MRSA bacteremia (5–7). Certain genetic elements, such as USA type and the presence of a dysfunctional accessory gene regulator (*agr*), may also affect survival rates (8–10). However, which factor influences the outcomes of interest to the greatest extent, taking into account all elements of the host-pathogen-treatment triad?

To answer this question, we set out to define the interplay between host, pathogen, and antimicrobial treatment when determining not only 30-day in-hospital mortality rates but also medium- and long-term mortality rates. In this way, the most relevant interventions can be developed and can be undertaken at specific times during the course of the bacteremia to yield the greatest impact on mortality rates.

## RESULTS

A total of 273 distinct patients met our inclusion criteria. All MRSA isolates were available for further study. The mean age of the entire cohort was 51.9 ± 15.9 years, and the majority (69.9%) of patients were male (Table 1). Only 26.3% of the patients were known carriers of MRSA, one-quarter used intravenous drugs, and 42% had recent health care exposure. The sources of infection varied, with close to one-half of the cases of bacteremia being secondary to endocarditis or skin and soft tissue infections. More than one-third of the patients were acutely ill, requiring intensive care unit (ICU) stays. Vancomycin represented the empirical drug of choice (97.1%), and appropriate antibiotics were administered in 86.4% of the cases. With both automated systems and broth microdilution assays, the median vancomycin MIC was 1 μg/ml (automated system interquartile range [IQR], 1 to 1 μg/ml; broth microdilution assay IQR, 0.5 to 1 μg/ml). USA100 and USA300 represented the dominant strains, and more than one-half of the isolates expressed Panton-Valentine leukocidin (PVL).

**TABLE 1:** Descriptive statistics for the entire cohort of 273 patients

Variable^*a*^	Finding
Age (mean ± SD) (yr)	51.9 ± 15.9
Male (no. [%])	191 (69.9)
Race (no. [%])	
White	110 (40.3)
Hispanic	103 (37.7)
Comorbidities (no. [%])	
Malignancy^*b*^	36 (13.2)
Alcoholism	39 (14.3)
Liver disease	50 (18.3)
ESRD/hemodialysis	30 (11)
Immunosuppression^*c*^	32 (11.7)
McCabe score result (no. [%])	
Nonfatal	185 (67.7)
Ultimately fatal	8 (2.9)
Rapidly fatal	80 (29.3)
IVDU (no. [%])	68 (24.9)
Health care exposure (no. [%])^*d*^	114 (41.8)
Source of bacteremia (no. [%])	
Endocarditis	59 (21.8)
Pneumonia	32 (11.7)
SSTI	73 (26.7)
CVC	32 (11.7)
Pitt bacteremia score (mean ± SD)	1.8 ± 2.6
ICU stay (no. [%])	99 (36.3)
Antibiotic treatment (no. [%])	
Appropriate antibiotics	235 (86.4)
Vancomycin	265 (97.1)
Linezolid	51 (18.7)
Daptomycin	53 (19.4)
Duration of antibiotic therapy (median [IQR]) (days)	32 (15–42)
Vancomycin MIC (median [IQR]) (μg/ml)	1 (1–1)
hVISA (no. [%])	1 (0.4)
PFGE result (no. [%])	
USA100	83 (33.3)
USA200	6 (2.4)
USA300	155 (62.3)
USA400	2 (0.8)
USA500	2 (0.8)
USA1200	1 (0.4)
PVL-expressing strain (no. [%])	88 (55.7)
Accessory gene regulator (no. [%])	
*agr* group I	94 (64.8)
*agr* group II	48 (33.1)
*agr* group III	3 (2.1)
*agr* loss of function (%)	14.3
MRSA carrier (no. [%])	67 (26.3)
Septic shock (no. [%])	27 (9.9)
Mechanical ventilation (no. [%])	58 (21.3)

a Abbreviations: SD, standard deviation; CVC, central venous catheter; ESRD, end-stage renal disease; hVISA, heterogeneous vancomycin-intermediate Staphylococcus aureus; IVDU, intravenous drug use; SSTI, skin and soft tissue infection.

b Malignancy had to be active at the time of bacteremia and not remote or in remission.

c Immunosuppression included bone marrow (3 patients [9%]) or solid organ transplantation (1 patient [3%]), AIDS (14 patients [44%]), use of prednisone at >10 mg for >14 days prior to infection (8 patients [25%]), and nontransplant immunosuppressive therapy (6 patients [19%]).

d Previous health care exposure within 90 days.

The median follow-up period was 493 days (IQR, 53 to 1,455 days). When patients who died during the first 30 days were excluded, the median follow-up period was 779 days (IQR, 251 to 1,761 days). Based on the Centers for Disease Control and Prevention (CDC) National Death Index (NDI) database and our data, we were able to ascertain the survival status for 97.4% of the patients at the end of the follow-up period.

The 30-day mortality rate was 18.7%, and the 30- to 90-day mortality rate was 7.7%. Death occurred for 59 patients (21.6%) after 90 days, for an overall mortality rate of 48%. The median time to death was 56 days (IQR, 14 to 291 days). We compared three subgroups of patients, i.e., 30-day, 30- to 90-day, and >90-day deaths (Table 2). Patients who died during the first 30 days were more likely to be older and to be sicker upon presentation, as evidenced by ICU stays, mechanical ventilation, and septic shock. The infections were most commonly caused by USA100 strains with *agr* group I. Patients who died between 30 and 90 days were more likely to have cancer, to be undergoing hemodialysis, and to have been exposed to the health care system. Those patients were also likely to have been started on vancomycin but then switched to daptomycin and linezolid. The third group, with late deaths (at >90 days), was composed of younger patients with less severe initial diseases who had bacteremia secondary to skin and soft tissue infections. Although the results were not statistically significant, infections were more commonly caused by USA300 strains and the presence of PVL was more prevalent in this group.

**TABLE 2:** Characteristics of patients who died <30 days, 30 to 90 days, or >90 days after the bacteremia index episode

Variable^*a*^	Patients who died in first 30 days (*n* = 51)	Patients who died between 30 and 90 days (*n* = 21)	Patients who died after 90 days (*n* = 59)	*P*
Age (mean ± SD) (yr)	61.5 ± 16	58.1 ± 17.6	50.1 ± 14.1	0.001
Male (no. [%])	33 (64.7)	14 (66.7)	41 (69.5)	0.866
Race (no. [%])				
White	23 (45.1)	9 (42.9)	20 (33.9)	0.464
Hispanic	15 (29.4)	5 (23.8)	25 (42.4)	0.195
Comorbidities (no. [%])				
Malignancy^*b*^	13 (25.5)	7 (33.3)	3 (5.1)	0.002
Alcoholism	11 (21.6)	2 (9.5)	8 (13.6)	0.352
Liver disease	16 (31.4)	4 (19.1)	13 (22)	0.413
IVDU	11 (21.6)	3 (14.3)	14 (23.7)	0.662
ESRD/hemodialysis	7 (13.7)	4 (19.1)	6 (10.2)	0.571
Immunosuppression	4 (7.8)	3 (14.3)	10 (17)	0.359
Health care exposure (no. [%])^*c*^	27 (52.9)	17 (81)	20 (33.9)	0.001
McCabe score result (no. [%])				0.010
Nonfatal	18 (35.3)	9 (42.9)	39 (66.1)	
Ultimately fatal	6 (11.8)	1 (4.8)	1 (1.7)	
Rapidly fatal	27 (52.9)	11 (52.4)	19 (32.2)	
MRSA carrier (no. [%])	15 (31.3)	8 (38.1)	16 (28.1)	0.696
Source of bacteremia (no. [%])				
Infective endocarditis	13 (26)	5 (23.8)	10 (17)	0.499
Pneumonia	9 (17.7)	3 (14.3)	4 (6.8)	0.211
SSTI	8 (15.7)	4 (19.1)	19 (32.2)	0.109
CLABSI	6 (11.8)	4 (19.1)	8 (13.6)	0.716
Acuity of presentation				
Pitt bacteremia score (median [IQR])	3 (1–7)	1.5 (0–3)	1 (0–2)	<0.001
ICU stay (no. [%])	37 (72.6)	11 (52.4)	16 (27.1)	<0.001
Septic shock (no. [%])	15 (29.4)	2 (9.5)	3 (5.1)	0.001
Mechanical ventilation (no. [%])	26 (51)	6 (28.6)	8 (13.6)	<0.001
Antibiotic treatment (no. [%])				
Appropriate antibiotics	42 (82.4)	18 (85.7)	53 (89.8)	0.523
Vancomycin	48 (94.1)	21 (100)	58 (98.3)	0.3
Linezolid	6 (11.8)	7 (33.3)	14 (23.7)	0.088
Daptomycin	8 (15.7)	6 (28.6)	7 (11.9)	0.2
PFGE result (no. [%])				0.144
USA100	24 (55.8)	8 (44.4)	17 (29.8)	
USA200	1 (2.3)	1 (5.6)	2 (3.5)	
USA300	17 (39.5)	9 (50)	38 (66.7)	
PVL-expressing strain (no. [%])	11 (37.9)	2 (16.7)	24 (60)	0.018
Accessory gene regulator (no. [%])				0.731
*agr* group I	15 (62.5)	5 (45.5)	18 (56.3)	
*agr* group II	9 (37.5)	6 (54.6)	13 (40.6)	
*agr* group III	0	0	1 (3.1)	
*agr* loss of function (no. [%])	6 (11.8)	2 (9.5)	6 (10.2)	0.855
hVISA (no. [%])	1 (2.2)	0	0	0.436
Vancomycin MIC (median [IQR]) (μg/ml)^*d*^	1 (1–1)	1 (1–1)	1 (1–1)	0.657

a SD, standard deviation; ESRD, end-stage renal disease; hVISA, heterogeneous vancomycin-intermediate Staphylococcus aureus; IVDU, intravenous drug use; SSTI, skin and soft tissue infection; CLABSI, central-line-associated bloodstream infection. McCabe scores, PFGE strain, and accessory gene regulator were treated as categorical variables.

b Malignancy had to be active at the time of bacteremia and not remote or in remission. The proportions of patients receiving chemotherapy at the time of bacteremia were 50% in the first group, 57% in the second group, and 66.7% in the third group.

c Previous health care exposure within 90 days.

d Vancomycin MICs were obtained using the Vitek2 and BD Phoenix automated systems.

Similar findings were demonstrated with Cox regression models. Thirty-day death was influenced mainly by host factors, both acute and chronic, such as age, cancer, liver disease, ICU stay, and Pitt bacteremia score (Table 3). Unresolved fever and unresolved leukocytosis were also significant predictors of 30-day death. Pitt bacteremia scores had prolonged effects on mortality rates, also affecting medium-term mortality rates (30 to 90 days), along with health care exposure and cancer (Table 4). Treatment duration of >4 weeks had a strong protective effect regarding medium-term death. This variable was treated as a dichotomous variable and was considered only for patients who survived past 30 days. Interestingly, admission leukocytosis was protective regarding long-term death, while increasing age was a significant risk factor (Table 5). The individual and collective results of each of the three models (0 to 30 days, χ^2^ = 9.83, *P* = 0.28; 30 to 90 days, χ^2^ = 2, *P* = 0.76; >90 days, χ^2^ = 0.49, *P* = 0.78) confirmed the assumption of proportional hazards.

**TABLE 3:** Univariable and multivariable Cox proportional-hazard models for 30-day death^*a*^

Predictor	HR (95% CI)		*P*
	Univariable analysis	Multivariable analysis	
Age	1.05 (1.03–1.07)	1.11 (1.07–1.15)	<0.001
Malignancy	2.3 (1.2–4.5)	2.5 (1.1–6)	0.033
Liver disease	2.3 (1.2–4.1)	4 (1.8–8.7)	0.001
Pitt bacteremia score	1.3 (1.2–1.4)	1.2 (1.1–1.3)	0.003
Septic shock	5.2 (2.8–9.5)	3.2 (1.4–7.5)	0.007
ICU stay	6 (3.2–11.4)	2.7 (1.2–6)	0.014
Time to leukocytosis normalization	8.4 (4.8–14.8)	2.4 (1.1–5)	0.024
Unresolved fever	10.2 (4.5–23.1)	9.7 (2.8–33.7)	<0.001

a USA300 strain, PVL, heterogeneous vancomycin-intermediate Staphylococcus aureus, history of lung disease, and history of heart failure all had *P* values of <0.1 in univariable analyses and were candidates for the multivariable analysis.

**TABLE 4:** Univariable and multivariable Cox proportional-hazard models for death between 30 and 90 days^*a*^

Predictor	HR (95% CI)		*P*
	Univariable analysis	Multivariable analysis	
Health care exposure	7.3 (2.5–21.6)	8.3 (2.4–29)	0.001
Malignancy	4.6 (1.9–11.4)	3.6 (1.4–9.4)	0.007
Pitt bacteremia score	1.2 (1–1.4)	1.3 (1.04–1.5)	0.019
4-wk antibiotic treatment	0.3 (0.1–0.7)	0.3 (0.1–0.7)	0.006

a Prior exposure to quinolones, number of antibiotics, history of congestive heart failure, weight, intravenous drug use, vancomycin level, cancer, Pitt score, age, and prior β-lactam use all had *P* values of <0.1 in univariable analyses and were candidates for the multivariable analysis.

**TABLE 5:** Univariable and multivariable Cox proportional-hazard models for death after 90 days^*a*^

Predictor	HR (95% CI)		*P*
	Univariable analysis	Multivariable analysis	
Age	1.02 (1–1.04)	1.02 (1.01–1.04)	0.036
Admission leukocytosis	0.56 (0.33–0.94)	0.53 (0.31–0.9)	0.019

a History of congestive heart failure, HIV, and osteomyelitis as the source of infection all had *P* values of <0.1 in univariable analyses and were candidates for the multivariable analysis.

**FIG 1:**
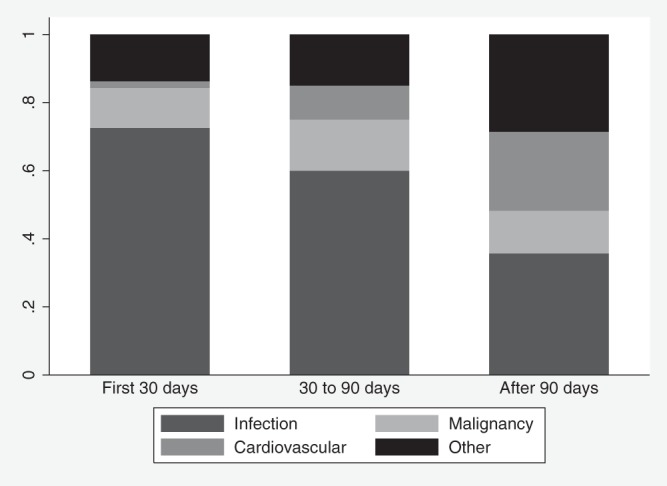
Causes of death among patients with MRSA bacteremia, according to mortality period.

Causes of death varied across our three groups. Infection represented the most frequent cause of death for all three time frames; proportions ranged from 72.6% in the first 30 days and 60% between 30 and 90 days to 35.7% at >90 days (*P* = 0.003) (Fig. 1). The contribution of malignancy to mortality rates remained stable at ∼10%. Cardiovascular causes increased from 2% to 23.2% for deaths that occurred >90 days after admission (*P* = 0.003).

During the follow-up period, 72 patients (26.4%) registered 139 readmissions. The majority of patients (*n* = 60) had 1 or 2 readmissions. Overall, 64/72 patients (88.9%) were readmitted after 90 days. Infectious problems were responsible for 51% of the readmissions, 51.4% of which were caused by MRSA.

Risk factors for readmissions caused by MRSA infections were the presence of prosthetic devices (subhazard ratio [sHR], 5.07 [95% confidence interval [CI], 1.8 to 815.7]; *P* = 0.003) and the time to normalization of the white blood cell (WBC) count (sHR, 1.05 [95% CI, 1.02 to 1.08]; *P* = 0.002).

## DISCUSSION

Our results describe host, pathogen, presentation severity, and treatment characteristics associated with three different patient trajectories, i.e., death within 30 days, death between 30 and 90 days, and death after 90 days. In our study, patients' attributes, including age, comorbidities (such as malignancy), and the acuity of presentation, shaped the trajectory in MRSA bloodstream infections. Treatment duration of >4 weeks had a protective effect regarding medium-term death. Although genetic markers such as USA type and *agr* had higher rates in different mortality periods, these associations were not statistically significant.

In our study, age significantly contributed to short- and long-term deaths, while cancer was a predictor of 30-day and 30- to 90-day deaths. Liver disease also affected short-term mortality rates. Multiple studies have emphasized the importance of age and comorbidities as predictors of poor outcomes in MRSA bacteremia (5, 6, 11). Older patients not only are at higher risk for acquiring and suffering from invasive MRSA infections but also are more likely to succumb to the infectious process. A robust WBC response, the opposite of a senescent immune system, had a protective effect regarding long-term death in our study. Younger age (51.9 years in our group versus older than 70 years in other cohorts) and fewer comorbidities could explain the lower short-term mortality rate in our cohort (7, 9).

Along with host attributes, the host-pathogen interactions that determine the severity of the acute presentation have also been repeatedly connected with short-term outcomes (12). It is interesting to note that, in our study, the Pitt bacteremia score, a validated marker of the severity of illness in bloodstream infections, affected also medium-term mortality rates. This could be explained by the protective effect of the aggressive ICU care that now pushes death into the medium term.

Various S. aureus genetic elements (e.g., clonal type, *agr* dysfunction, and the presence of PVL) have been linked to increased virulence, persistence of infection, and lower efficacy of antimicrobials. The proven clinical consequences are still a matter of debate. In our study, the predominant strain was USA300, which in general causes community-acquired infections, particularly skin and soft tissue infections. Overall, USA300 is becoming the most common strain in the United States (13–15). This clone is considered more virulent, but its impact on mortality rates remains unclear, especially with increasing contributions to hospital-acquired infections (9, 14, 15). In a population-based surveillance study in Atlanta, Georgia, USA300 clones had higher mortality rates than did USA100 strains, and the clonal strain remained a significant predictor of death in the multivariable analysis (hazard ratio [HR], 1.6 [95% CI, 1.2 to 2.2]) (9). The study did not include treatment factors, and age was the major negative confounder for USA300. In our study, USA300 did not significantly influence short-, medium-, or long-term mortality rates, but it was present more in the group of patients who died after 90 days and had skin and soft tissue infections as the source of bacteremia. This subgroup of patients was also distinguished by the presence of PVL, which helps to establish an aggressive infection but does not seem to influence mortality rates in patients with bacteremia (16, 17). In a study of 89 strains collected in a multicenter trial, it was noted that USA300 strains and strains constitutively expressing PVL were associated with better outcomes (17).

The accessory gene regulator controls the production of many virulence genes, including exotoxins and quorum-sensing peptides. Past *in vitro* studies have associated *agr* dysfunction with delayed microbial clearance and reduced vancomycin efficacy (18). However, the results are not so pronounced *in vivo* (19). The *agr* type and functionality did not differentiate between simple MSSA bacteremia and complicated MSSA bacteremia (defined as end-organ spread, endocarditis, septic thrombophlebitis, or persistent bacteremia) in a Spanish trial (20). In another study, *agr* dysfunction affected 30-day mortality rates only in the sickest group of patients, with modified acute physiology scores of ≥28. This effect was maintained after adjustment for comorbidities, age, vancomycin MIC, and vancomycin administration (8). In our study, *agr* dysfunction was encountered less than previously reported in the literature. One hypothesis could be the lack of selective pressure caused by antibiotics, since our cohort was younger and with less comorbidities than usual.

Timely appropriate antibiotic therapy remains the cornerstone of treatment for all infections, and MRSA bacteremia is no exception, although the cutoff time varies from study to study (7). The benefit of appropriate antibiotics is even greater in patients with septic shock. The duration of antimicrobial treatment has always been debated (2 to 4 weeks versus 4 to 6 weeks), and different criteria have been published. Similar to findings for complicated bacteremia (21), an antimicrobial course of at least 4 weeks must be employed to decrease medium-term mortality rates, according to our results. Along with timing and duration, the choice of antimicrobial has been studied. Our cohort had vancomycin MICs in the susceptible range, making vancomycin the drug of choice. The first measured trough was 17.1 μg/ml, on average, and only the duration of therapy affected the outcomes of interest.

Complex interactions between the host, pathogen, and antimicrobials evolve continuously, as the antibiotic can alter the microbe's virulence and the microbe phenotype can in turn affect the host's response and the response to therapy. Moreover, S. aureus can cause metastatic infections when mixed bacterial populations (e.g., *agr* functional and *agr* defective) can coexist. Different aspects of this interaction may play a role in initiating versus propagating and maintaining the infectious process.

In general, most infectious diseases evolve acutely, but we are now discovering the long-term repercussions of pneumonia and bloodstream infections, even after complete recovery from the initial episode. Cardiovascular consequences and decreased long-term survival rates have been illustrated for community-acquired pneumonia (22). Accordingly, more emphasis is being placed on the downstream effects of bloodstream infections. In a very large recent study by Nelson et al. (23), hospital-acquired MRSA infections led to increased risk of attributable deaths at both 30 and 90 days, compared to noninfected controls, using a propensity score algorithm (relative risk [RR], 2.2 [95% CI, 2 to 2.5]; *P* < 0.001). Moreover, the trend endured even for cultures from nonsterile sites, indicating that even colonized patients carry a greater risk of death (23). The same group conducted a similar study with the Veterans Affairs population, showing that hospital-acquired MRSA infections increased the risk of death at 1 year by 46% (24). Previous literature findings indicated additional long-term deaths with MRSA bloodstream infections (25–27) but did not include bacterial virulence factors. Other studies have emphasized the importance of the clinical entity over microbiological traits only for short-term outcomes (28).

Our study has several limitations worth mentioning. It is an observational study performed in a single center, focusing solely on MRSA bacteremia outcomes. Although we had extensive data collection, masked confounders might have been missed. The NDI provided data on survival status, but data on the cause of death might be incomplete. A randomized trial looking at attributes of patients and microbes obviously would be impossible. Nevertheless, our database covered all known potential contributors to short-, medium-, and long-term mortality rates in a large cohort of patients with MRSA bacteremia. We did not have information about consultations by infectious diseases specialists. The large number of tests performed without adjustment of the *P* values increases the likelihood that some of the test results are false-positive results. Over the course of the study, microbiological methods and treatment options have changed, which might have affected our results.

To our knowledge, this is the first study to incorporate all known risk factors (host, pathogen, and treatments) and to examine their effects on short-, medium-, and long-term mortality rates. Our results could be helpful in devising new targeted approaches for MRSA bloodstream infections. In acute settings, markers such as pentraxin 3, cell-free DNA, and serum interleukin 10 (IL-10) levels may classify patients as being at risk for poor immediate outcomes, and aggressive resuscitative measures and timely antibiotic treatment should be started (29, 30). Admission leukocytosis is a readily available variable that could modify the intensity of the applied treatments. Since comorbidities are unlikely to be altered, screening and decolonization practices for patients at high risk (e.g., cancer patients) may be studied in the future. The duration of therapy and the prevention of relapses may alter medium- and long-term mortality rates. Interventions can be directed at decreasing the rates of reinfection or addressing the cardiovascular consequences of MRSA bloodstream infections.

In conclusion, host characteristics affect short-, medium-, and long-term mortality rates for MRSA bloodstream infections more than do pathogen genetic markers and treatment factors. Interventions should consider the trajectory of various subpopulations affected by MRSA bloodstream infections.

## MATERIALS AND METHODS

### Setting and patients.

The study took place at the University of New Mexico (UNM) Health Sciences Center (HSC), a tertiary-care academic referral hospital, and was approved by the institutional review board. The need for consent was waived, given the retrospective nature of the study.

All MRSA strains isolated from blood cultures obtained from adult patients who were admitted between July 2002 and August 2013 were entered in our database. Identification of all bacterial isolates was performed by the UNM HSC reference laboratory (TriCore Reference Laboratories, Albuquerque, NM). The included patients were adult patients who were admitted to the UNM HSC during the study period and had blood cultures positive for MRSA. All episodes of bacteremia were recorded, but only the initial episode was used in the analyses.

For all patients, clinical data, including demographic data, McCabe scores, and data on comorbidities of interest, MRSA carriage, prosthetic devices and catheters, the source of bacteremia, and markers of severity of acute presentation (ICU stay, mechanical ventilation, shock, and Pitt bacteremia score), were recorded. We also assessed laboratory, imaging, and treatment variables, including drugs used, treatment duration, vancomycin level troughs, changes in antimicrobial therapy, and infected device removal and source control. The McCabe score, as modified by Soriano et al. (31), classifies patients based on the severity of the underlying disease, i.e., rapidly fatal (death expected to occur in <3 months), ultimately fatal (death expected to occur between 3 months and 5 years), or nonfatal (32). The score has been validated for MRSA bacteremia (31). Malignancy was defined as active cancer, with or without chemotherapy. Immunosuppression consisted of transplantation, AIDS, use of corticosteroids (equivalent of >10 mg of prednisone daily for >2 weeks), or use of nontransplant immunosuppressive medications. The Pitt bacteremia score takes into account clinical variables such as fever, hypotension, mechanical ventilation, cardiac arrest, and mental status during the initial 48 h, and scores correlate with mortality rates (6). Health care exposure was defined as home intravenous antibiotic therapy or infusion clinic attendance 30 days prior to infection, hospital admission for ≥2 days within the past 90 days, hemodialysis 30 days prior to infection, or residence in a nursing home or long-term-care facility. Prior antibiotic use had to occur within 30 days before the index admission. Appropriate antimicrobial therapy was defined as initiation of at least one antimicrobial to which the bacterium was susceptible *in vitro* within the first 24 h after collection of the index blood culture.

### Microbiology.

The UNM HSC reference laboratory collaborates with TriCore Reference Laboratories for microbiology tests. Positive blood cultures were processed and susceptibility patterns were determined using the BD Phoenix system. *In vitro* vancomycin MIC determinations were analyzed by automated dilution testing, according to Clinical and Laboratory Standards Institute (CLSI) guidelines (33), with one of two microbial identification systems, depending on the year of collection, i.e., (i) Vitek2 (2002 to 2009) or (ii) BD Phoenix (2009 to 2013). Subcultures of all bacterial isolates were collected and stored at −80°C until further use. MICs were then confirmed in the UNM College of Pharmacy research laboratory with the broth microdilution technique, following methods described by the CLSI (33).

Bacterial isolates were characterized for MRSA strain type by pulsed-field gel electrophoresis (PFGE) (see the supplemental material) (34). The expression of PVL was determined by real-time PCR as described by Johnsson et al. (35), with slight modifications (see the supplemental material). Isolates were characterized for accessory gene regulator (*agr*) group using a previously described quantitative multiplex PCR assay (36), and *agr* function was assessed semiquantitatively using a δ-hemolysis assay, as described previously (see the supplemental material) (37). Glycopeptide resistance detection (GRD) E-tests (bioMérieux, Piscataway, NJ) were used for heteroresistance testing (38).

### Outcomes and follow-up monitoring.

Unresolved fever and leukocytosis were defined as persistence beyond 5 days after the initiation of appropriate antimicrobial treatment. Outcomes of interest were short-term deaths (defined as all-cause deaths within 30 days), medium-term deaths (deaths between 30 and 90 days), and long-term deaths (deaths after 90 days). We also looked at the timing and causes of readmission throughout the study period. We determined survival as of December 2014 by reviewing the UNM HSC electronic medical records system. Cases with missing records were sent to the NDI, a CDC centralized database of information on death records. Determination of survival status according to the NDI records was assessed and validated previously (39).

### Statistical methods.

Means and standard deviations were reported for continuous variables if data were normally distributed; otherwise, medians and IQRs were reported. These data were analyzed with analysis of variance (ANOVA) or the Kruskal-Wallis test. Proportions were compared using the χ^2^ test or Fischer's exact test, as needed. Cox regression models were used to identify independent risk factors for all-cause deaths. Candidate variables with *P* values of <0.1 in Cox regression univariable analyses based on the relevant characteristics described were included in the multivariable models. For the 30-day mortality rates, all patients were included in the analyses, and patients surviving past 30 days were right censored at that point. For the period of 30 to 90 days, we excluded patients who had died prior to 30 days, and patients surviving past 90 days were right censored at 90 days. For the last period, >90 days, patients who had died previously were eliminated, and we compared only survivors versus nonsurvivors past 90 days. Tested interactions were based on biological plausibility and medical literature findings. Therefore, we examined and created interaction indicators for each of the following combinations: for the first period, age and Pitt bacteremia score, age and unresolved fever, age and time to WBC count normalization, liver disease and septic shock, liver disease and ICU stay, and malignancy and ICU stay; for the second period, health care exposure and malignancy; for the third period, age and increased WBC count. None of the interaction terms was significant after being included in the final model as a variable.

The assumption of proportional hazards was tested by using log(time) interactions in all models and goodness of fit by plotting Schnell residuals. For readmission rates, we used competing risk methods, as described by Fine and Gray (40). Statistical analyses were performed using Stata 13.1 SE (Stata Corp., College Station, TX).

## Supplementary Material

Supplemental material
