# Supplementary material for: Host-Pathogen-Treatment Triad: Host Factors Matter Most in Methicillin-Resistant Staphylococcus aureus Bacteremia Outcomes
Source: Antimicrob Agents Chemother. 2018 Jan 25;62(2):e01902-17. doi: 10.1128/AAC.01902-17 (PMC5786753; doi:10.1128/AAC.01902-17)
Supplement: Supplemental material [file supp_62_2_e01902-17__index.html]

Supplemental material 

# Host-Pathogen-Treatment Triad: Host Factors Matter Most in Methicillin-Resistant Staphylococcus aureus Bacteremia Outcomes

## Supplemental material

- Supplemental file 1 -

  Supplemental methods and figures

  PDF, 117K
