# Supplementary material for: Host-Pathogen-Treatment Triad: Host Factors Matter Most in Methicillin-Resistant Staphylococcus aureus Bacteremia Outcomes
Source: Antimicrob Agents Chemother. 2018 Jan 25;62(2):e01902-17. doi: 10.1128/AAC.01902-17 (PMC5786753; doi:10.1128/AAC.01902-17)
Supplement: Supplemental material [file AAC.01902-17_zac002186886s1.pdf]

Supplement 1:

Pulsed field gel electrophoresis (PFGE).

Isolates were embedded in agarose plugs and lysed in situ, and genomic DNA was digested with SmaI restriction endonuclease (New England Biolabs, Beverly, MA). The restriction fragments were resolved into a pattern of discrete bands in a 1% SeaKem Gold agarose gel by switching the current direction starting at 2 seconds and finishing at 40 seconds, using a linear ramping factor and a total run time of 16.5 hours. DNA fragment patterns were visualized by ethidium bromide staining and analyzed using the GelCompar II V3.0 imaging software.

Panton-Valentine leukocidin (PVL) expression:

Oligonucleotide primers have been designed to amplify a region spanning the last section of lukS-PV and the beginning adjacent portion of lukF-PV [4] (5'-GCTGGACAAAACTTCTTGGAATAT-3' and 5'-GATAGGACACCAATAAATTCTGGATTG-3'; correspond to positions 2666-2690 and 2749-2723 of the PVL sequence, respectively). PCR amplification (25 µl) contained 0.25 ng of DNA, 500 nM each forward and reverse primer, 12.5 ml iQ™ SYBR green Supermix (Biorad), and 9.5 ml sterile water. Reactions are initiated at 95°C for 10 minutes; followed by 35 cycles of 30 seconds at 95°C and 2 minutes at 60°C. Melt curve analysis were used to confirm presence of a single product. All samples were tested in triplicate and results for each run averaged. PVL-positive and PVL-negative *S. aureus* isolates (MW2 and CCUG 35601, respectively) were analyzed as controls in each amplification run.

Individual isolates were cultured in tryptic soy broth (TSB) for 2 hours at 37°C with mixing. Zirconia beads and a BeadBeater-type homogenizer (BioSpec, Bartlesville, Oklahoma, USA) were used to extract DNA from bacterial pellets resuspended in 10 mM Tris, pH 8.0, 1 mM EDTA. DNA from each isolate was amplified by qPCR using primers and probes corresponding to variable regions of *agr*C specific for each *agr* group. Each amplification assay included known *agr* group internal controls.

Accessory gene regulator (*agr*) typing by PCR.

Accessory gene regulator (*agr)* function was semi-quantitatively assessed using a δ-

hemolysis assay as previously described.[6] Individual isolates were streaked outwardly from a beta-lysin disk (Remel, Lenexa, KS) that was placed on a Muller-Hinton Agar (MHA) with 5% sheep blood agar plate. Plates were incubated at 37°C for 24 hours and read independently by two investigators. δ-hemolysin was considered present if there was an observed synergistic hemolysis within the zone of overlap between the beta-lysin hemolysis from the disk and the δ-hemolysis from the test strain. Such observed hemolysis was interpreted as presence of *agr* function. Strains RN6607 and RN9120 were utilized as positive and negative controls, respectively.

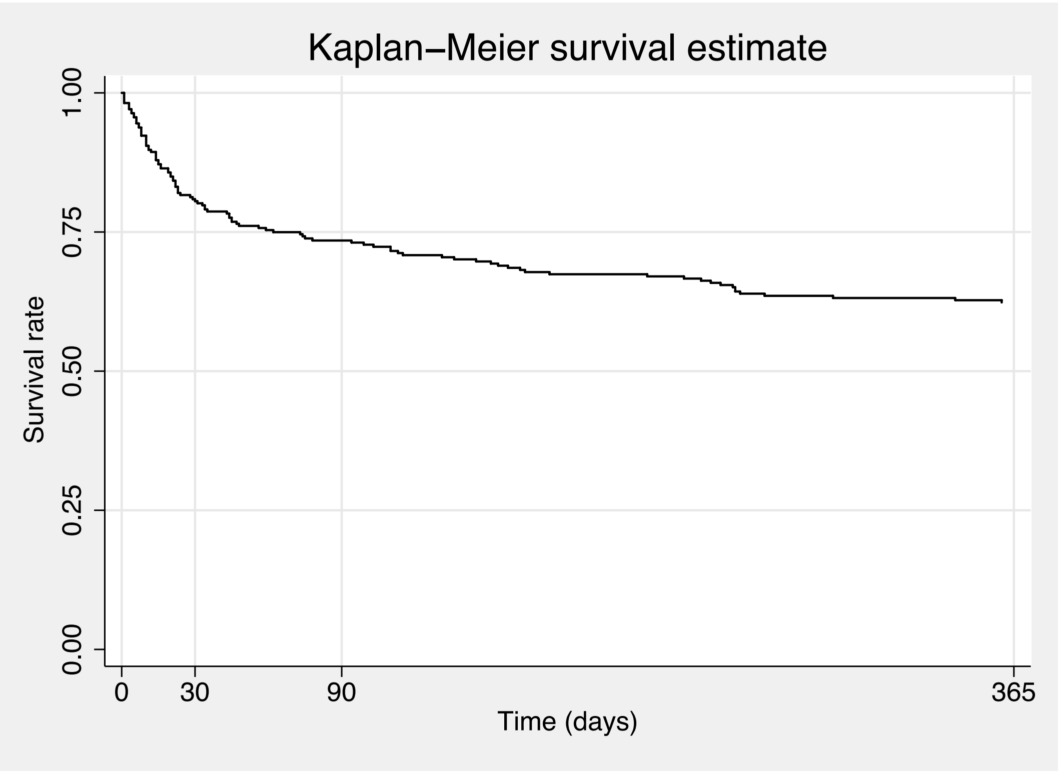

Supplementary Figure 1. Kaplan Meier survival curve for the first 1 year.

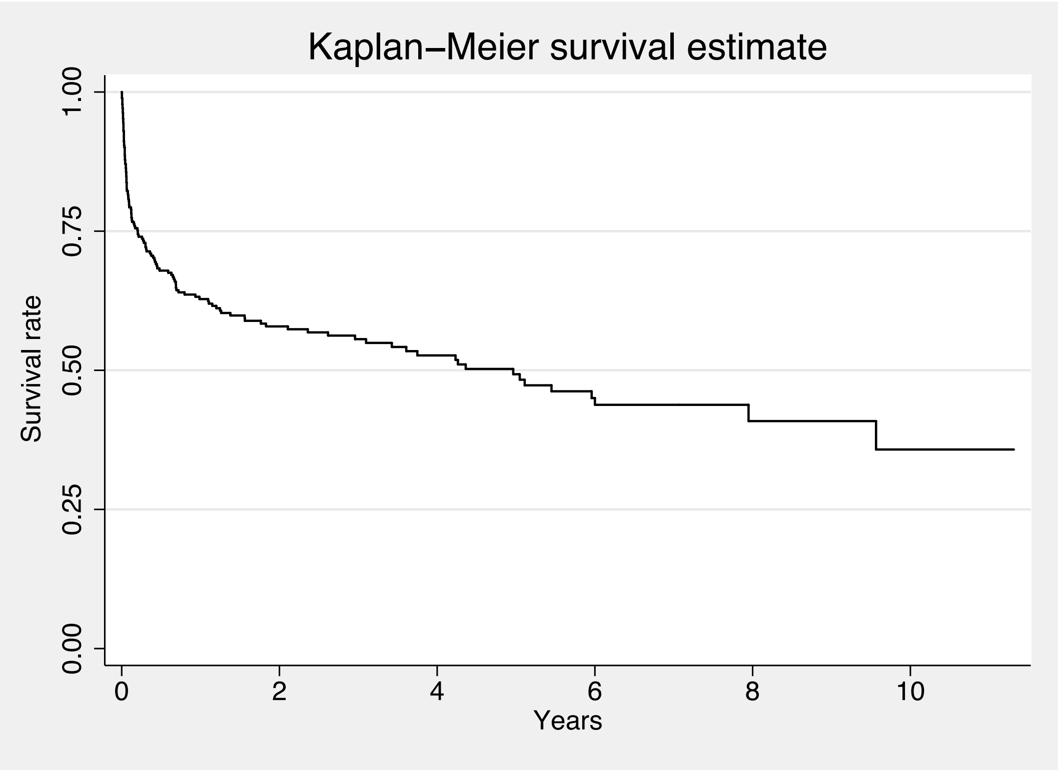

Supplementary Figure 2. Kaplan Meier survival curve for the follow up period.
